# The Association Between Air Pollution Exposure and White Blood Cell Counts: A Nationwide Cross-Sectional Survey in South Korea

**DOI:** 10.3390/jcm13237402

**Published:** 2024-12-05

**Authors:** Jihye Lee, Hee-Young Yoon

**Affiliations:** 1 Division of Pulmonary and Critical Care Medicine, Department of Internal Medicine, Soonchunhyang University Cheonan Hospital, Cheonan 31151, Republic of Korea; icujh@schmc.ac.kr; 2 Division of Allergy and Respiratory Diseases, Department of Internal Medicine, Soonchunhyang University Seoul Hospital, Seoul 04401, Republic of Korea

**Keywords:** air pollution, white blood cell count, systemic inflammation, particulate matter, nitrogen dioxide, epidemiologic studies

## Abstract

**Background:** The effect of air pollution, a major global health issue, on the immune system, particularly on white blood cell (WBC) counts, remains underexplored. **Methods:** This study utilized data from 54,756 participants in the Korean National Health and Nutrition Examination Survey to investigate the effects of short- (day of examination and 7-day averages), mid- (30- and 90-day averages), and long-term (one-, three-, and five-year averages) air pollutant exposure on WBC counts. We assessed exposure to particulate matter (PM_10_, PM_2.5_), sulfur dioxide (SO_2_), nitrogen dioxide (NO_2_), ozone (O_3_), and carbon monoxide (CO). **Results:** Linear regression with log-transformed WBC counts, adjusted for confounders, showed that PM_10_ was positively associated with long-term exposure, PM_2.5_ was negatively associated with short- and mid-term exposures, SO_2_ was consistently negatively associated with short- and mid-term exposures, NO_2_ and CO were positive across most periods, and O_3_ was negatively associated with short- and mid-term exposures. Logistic regression analysis confirmed these findings, showing that short- and mid-term exposure to PM_10_, PM_2.5_, and SO_2_ was negatively associated with the risk of belonging to the high-WBC group, while long-term exposure to PM_10_, PM_2.5_, NO_2_, and CO showed positive associations with risk. **Conclusions:** Our findings highlight the time- and pollutant-specific associations between air pollution exposure and WBC counts, underscoring air pollution’s potential impact on systemic inflammation.

## 1. Introduction

Air pollution, a major global health concern, has a significant impact on the immune system [1]. By 2050, approximately 70% of the world’s population will reside in cities, making air pollution a nearly universal issue [2]. This pollution, primarily arising from human activities such as industrial manufacturing, power plant operations, vehicle emissions, and fossil fuel burning, is linked to various health issues [3].

While the short- and long-term health effects of air pollution on respiratory and cardiovascular diseases are well documented [4,5], its impact on the immune system remains unclear. White blood cells (WBCs), or leukocytes, essential for both innate and adaptive immune responses, circulate in the blood to mediate inflammation and combat pathogens or injury [6]. Variations in WBC counts, influenced by factors like infection, trauma, and chronic conditions, often signal systemic inflammation and indicate a wide range of health states [7]. Elevated WBC counts, particularly at the higher end of the normal spectrum, are associated with an increased risk of both cardiovascular and non-cardiovascular mortality [8,9,10], suggesting their potential as biomarkers for underlying systemic inflammation and subclinical disease.

Air pollution can affect the immune system, potentially leading to systemic inflammation [11,12]. WBC count, a readily measurable marker, can serve as a surrogate indicator of systemic inflammation and the health effects of air pollution. Previous studies have reported an association between WBC count and short-term air pollution exposure [13,14,15,16,17,18,19,20,21,22,23,24], highlighting the profound influence of air pollution on immune system functions. However, most of these studies have focused on short-term effects and involved small sample sizes.

Therefore, this study aimed to examine the association between short-, mid-, and long-term air pollution exposure and WBC count through a comprehensive cross-sectional survey of a healthy Korean population.

## 2. Materials and Methods

### 2.1. Data Source

We used data from the Korean National Health and Nutrition Examination Survey (KNHANES), a comprehensive annual survey conducted by the Korea Disease Control and Prevention Agency to inform health policies and programs [25]. From 1998 to 2023, the KNHANES has improved its health statistics through continuous annual surveys, full-time field staff, an expansion of survey components, collaboration with academic societies for quality control, and periodic method revisions.

Data collection methods include health interviews, physical examinations, and nutritional surveys that cover socioeconomic status, smoking, alcohol use, physical activity, nutrition, comorbidities (e.g., obesity, hypertension, diabetes, pulmonary disease, dyslipidemia, and kidney disease), and oral health. Every five years, the KNHANES rotates specific topics, such as mental health, sleep, smoking, alcohol use, physical activity, nutrition, obesity, cardiovascular disease, indoor air quality, eye diseases, otolaryngologic diseases, sarcopenia, and osteoporosis.

Since 2021, linked air quality data from 2007 to 2020 has been included, measuring particulate matter 10 and 2.5 μm or less in diameter (PM_10_ and PM_2.5_), sulfur dioxide (SO_2_), nitrogen dioxide (NO_2_), ozone (O_3_), and carbon monoxide (CO), temperature, humidity, and other factors. These data cover a study population of 89,613 participants [26].

### 2.2. Study Population

**Figure 1:**
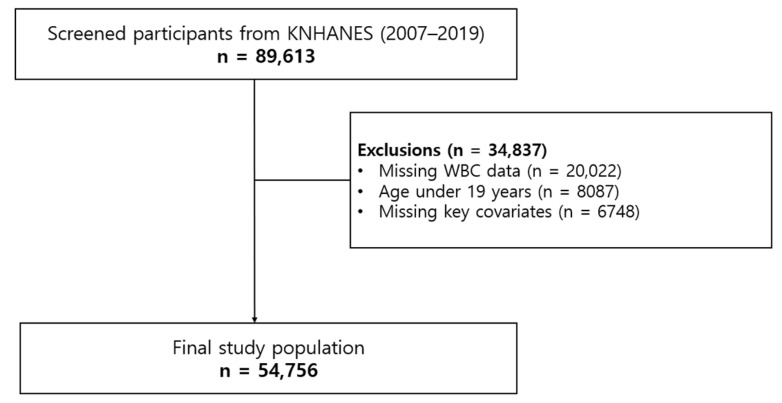
Flow chart of patient enrollment.

From the 89,613 participants in the KNHANES cohort (2007–2019), 34,837 were excluded: 20,022 due to missing WBC counts, 8087 for being under 19 years old, and 6748 due to missing key covariates (Figure 1). The final study population included 54,756 participants.

Informed consent was waived due to the use of anonymized data. This study was approved by the Institutional Review Board of the Soonchunhyang University Seoul Hospital (SCHUH 2023-08-002).

### 2.3. Clinical Data

Blood samples were collected in the morning after at least eight hours of fasting. Complete blood counts, including WBC, red blood cell, and platelet counts, were measured using XN-900 (Sysmex, Kobe, Japan).

Data on demographics, lifestyle habits, and physical characteristics were collected to explore the relationship between exposure to air pollution and inflammatory markers. Demographics included sex, age, smoking status, body mass index, educational level, household income, and walking days in a week. Education was categorized into four levels (less than elementary school, middle school, high school, and more than college) and income into quartiles. The variable walking days in a week was categorized into 8 levels, ranging from 0 days to daily walking.

### 2.4. Air Pollution Exposure Assessment

This study utilized a comprehensive database created using the Community Multiscale Air Quality model to integrate meteorological and air quality data [26]. This database integrates meteorological data (wind, temperature, and humidity fields) with emission quantities modeled for chemical composition and spatiotemporal distribution. The initial estimates used a 27 km grid resolution for East Asia and a 9 km resolution for the Korean Peninsula. Weather Research and Forecasting version 3.6.1 (Boulder, CO, USA) was employed for meteorological data modeling, while Sparse Matrix Operator Kernel Emissions version 2.7 (Chapel Hill, NC, USA) was used for emission data modeling [27].

Hourly measurements of PM_10_, PM_2.5_, SO_2_, NO_2_, O_3_, and CO were obtained from Air Korea (https://airkorea.or.kr/, accessed on 1 January 2023), operated by the South Korean Ministry of the Environment. This dataset also included daily averages of temperature, humidity, precipitation, wind direction, solar radiation, and surface pressure measured every 3 km. Additionally, air quality data encompassing PM_10_ and PM_2.5_ (measured every 1 km^2^) and gaseous pollutants like NO_2_, CO, SO_2_, and O_3_ (measured every 9 km^2^) were included. Data assimilation techniques refined initial estimates using observed values. The reanalysis results were validated through multiple regressions applied to satellite-observed aerosol optical depth data.

The KNHANES database was merged with the air quality data using the participants’ residential addresses linked using geocode units to ensure accurate exposure assessment.

Short-term exposure was defined as the level of exposure on the day of the examination and the 7-day (0–6 days) average. Mid-term exposure was defined as the 30-day and 90-day moving averages. Long-term exposure was defined as the average exposure over one, three, or five years. Exposure values were calculated as the arithmetic mean of the time-series data for each period.

### 2.5. Statistical Analysis

Continuous variables are presented as mean ± standard error or median (interquartile range), while categorical variables are presented as numbers (%).

Linear regression analyses were conducted using ln-transformed WBC counts as the dependent variable to investigate their association with air pollution levels [28,29]. The results were expressed as beta coefficients with 95% confidence intervals (CIs) and *p*-values. To improve interpretability, the regression coefficients and their corresponding confidence intervals were back-transformed and reported as percentage (%) changes, representing the relative change in WBC counts associated with a unit increase in air pollutant levels [30]. To account for potential confounding factors, univariate and multivariable analyses were performed, adjusting for covariates such as examination year, sex, age, income, education level, smoking status, physical activity, hemoglobin level, platelet count, and environmental factors including temperature, wind speed, humidity, precipitation, wind direction, solar radiation, and surface pressure. Correlations between air pollutants were assessed using Pearson correlation coefficients to explore potential interrelationships among pollutants.

To further evaluate the impact of air pollution on elevated WBC counts, defined as those exceeding the median value (5.91 × 10^3^/µL), binary logistic regression analyses were conducted in both univariate and multivariable frameworks. Given that air pollution levels did not follow a normal distribution, pollutant exposures were analyzed in terms of interquartile range (IQR) increases, ensuring consistency and comparability across all statistical models.

The analysis used data from the 2007–2019 KNHANES, applying sampling weights to account for its complex, stratified multistage design, adjusting for selection probabilities, non-response, and post-stratification. Details are provided in Supplementary Table S1. All analyses were performed using IBM SPSS 25.0 software (SPSS Inc., Chicago, IL, USA), Rex (version 3.6.1) (RexSoft, Seoul, Republic of Korea), and the R “survey” (version 4.1-1, Thomas Lumley, 2020, https://r-survey.r-forge.r-project.org/survey/, access on 1 November 2024) package (R version 4.3.2, Lucent Technologies, Murray Hill, NJ, USA). A two-tailed *p*-value of less than 0.05 was considered statistically significant.

## 3. Results

### 3.1. Participant Characteristics

The mean age of the 54,756 individuals was 49.78 years, and 43.2% (23,663) were male (Table 1). Income levels were relatively evenly distributed among participants, although the lowest income group represented the smallest proportion, at 24.1%. Regarding education levels, high school graduates formed the largest group, and 31.8% had received a college education or higher. Ever-smokers accounted for 38.3% of the participants. The mean and median (IQR) WBC counts were 6.15 ± 0.01 and 5.91 (2.1) × 10^3^/µL, respectively. The median (IQR) hemoglobin level was 13.9 (2.2) g/dL, and the median platelet level was 251 (74) × 10^3^/µL.

### 3.2. Air Pollutant Levels and Environmental Factors

The on-the-day mean concentrations of particulate matter, PM_10_ and PM_2.5_, were 50.06 ± 0.10 μg/m^3^ and 24.79 ± 0.05 μg/m^3^, respectively (Table 2). The on-the-day mean concentrations of gaseous pollutants SO_2_, NO_2_, CO, and O_3_ were 4.83 ± 0.01 ppb, 25.34 ± 0.06 ppb, 487.49 ± 0.92 ppb, and 26.66 ± 0.06 ppb, respectively. The median (IQR) air pollutant levels and the means for environmental factors on the examination day are shown in Supplementary Tables S2 and S3, respectively.

A Pearson correlation analysis showed strong positive correlations between PM_10_ and PM_2.5_ (r = 0.892, *p* < 0.001) as well as NO_2_ and CO (r = 0.709, *p* < 0.001) (Supplementary Table S4). O_3_ was negatively correlated with other pollutants, particularly NO_2_ (r = −0.462, *p* < 0.001) and CO (r = −0.407, *p* < 0.001).

### 3.3. Correlation Between WBC Count and Air Pollutants

For PM_10_, the unadjusted analysis showed a negative association with WBC counts for mid-term exposures (Table 3). In the adjusted analysis, significant negative associations were observed for 90-day exposure (−0.03%, 95% CI: −0.05–−0.01, *p* = 0.006). Long-term exposures demonstrated significant positive associations in the adjusted analysis, with WBC counts increasing by 0.09% (95% CI: 0.06–0.13, *p* < 0.001) for 1-year, 0.12% (95% CI: 0.08–0.15, *p* < 0.001) for 3-year, and 0.13% (95% CI: 0.08–0.17, *p* < 0.001) for 5-year exposures, with subtle changes.

For PM_2.5_, the unadjusted analysis revealed significant negative associations with WBC counts for short-term and mid-term exposures. The adjusted analysis showed negative associations for short-term (on-the-day: −0.02%, 95% CI: −0.04–0.00, *p* = 0.022; 7-day: −0.03%, 95% CI: −0.06–0.00, *p* = 0.020), mid-term (30-day: −0.07%, 95% CI: −0.11–−0.03, *p* < 0.001; 90-day: −0.12%, 95% CI: −0.16–−0.08, *p* < 0.001), and long-term exposures (5-year: 0.22%, 95% CI: 0.15–0.30, *p* < 0.001).

For SO_2_, both the unadjusted and adjusted analyses consistently showed significant negative associations with WBC counts for short-term exposures (on-the-day: −58.01%, 95% CI: −80.23–−10.82, *p* = 0.024; 7-day: −72.31%, 95% CI: −88.53–−33.14, *p* = 0.004; 30-day: −72.46%, 95% CI: −89.34–−28.81, *p* = 0.008) and mid-term exposure (90-day: −76.35%, 95% CI: −91.16–−36.72, *p* = 0.004).

For NO_2_, the unadjusted analysis showed no statistically significant associations with WBC counts. However, the adjusted analysis revealed positive associations across all exposure periods, including short-term (on-the-day: 38.46%, 95% CI: 15.29–66.30, *p* < 0.001; 7-day: 43.85%, 95% CI: 17.38–76.28, *p* < 0.001), mid-term (30-day: 49.89%, 95% CI: 20.63–86.25, *p* < 0.001; 90-day: 34.45%, 95% CI: 8.36–66.81, *p* = 0.007), and long-term exposures (1-year: 71.24%, 95% CI: 35.90–115.76, *p* < 0.001; 3-year: 55.73%, 95% CI: 23.30–96.70, *p* < 0.001; 5-year: 48.20%, 95% CI: 13.21–94.01, *p* = 0.004).

Similar to that for NO_2_, the unadjusted analysis for CO showed no statistically significant associations with WBC counts. However, the adjusted analysis revealed significant positive associations for all exposure periods except average for the 90-day period, with increases observed for short-term exposures (on-the-day: 1.93%, 95% CI: 0.74–3.14, *p* = 0.001; 7-day: 1.85%, 95% CI: 0.32–3.40, *p* = 0.017) and long-term exposures (1-year: 9.56%, 95% CI: 6.89–12.30, *p* < 0.001; 3-year: 10.08%, 95% CI: 7.43–12.80, *p* < 0.001; 5-year: 8.56%, 95% CI: 5.39–11.83, *p* < 0.001).

For O_3_, the unadjusted analysis showed significant positive associations with WBC counts for mid-term and long-term exposures. However, the adjusted analysis revealed significant negative associations for short-term exposures (on-the-day: −35.40%, 95% CI: −47.70–−20.21, *p* < 0.001; 7-day: −45.11%, 95% CI: −56.45–−30.81, *p* < 0.001), mid-term exposure (30-day: −40.69%, 95% CI: −54.61–−22.49, *p* < 0.001), and long-term exposure (5-year: −60.70%, 95% CI: −79.78–−23.60, *p* = 0.006).

### 3.4. Impact of Short-Term Air Pollutant Exposure on High-WBC Group

While PM_10_ exposure on day 0 did not show a significant association with WBC count in the unadjusted analysis, it was significantly associated with the low-WBC group in the adjusted analysis (OR = 0.976, 95% CI: 0.952–0.999, *p* = 0.045) (Table 4). Increased PM_2.5_ exposure on day 0 was associated with the low-WBC group in both the unadjusted and adjusted analyses (OR = 0.965, 95% CI: 0.941–0.988, *p* = 0.004). Similarly, high SO_2_ exposure on day 0 showed a negative association with the high-WBC group in both the unadjusted and adjusted analyses (OR = 0.979, 95% CI: 0.961–0.998, *p* = 0.027), while 7-day exposure showed a negative association only in the unadjusted analysis. No significant associations were observed for NO_2_, CO, or O_3_.

The ORs were calculated per interquartile range increase in the on-the-day and 7-day averages for each air pollutant: PM_10_—26.61 and 21.2 μg/m^3^; PM_2.5_—14.84 and 11.11 μg/m^3^; SO_2_—2.8 and 2.53 ppb; NO_2_—19.65 and 17.54 ppb; CO—229 and 192.26 ppb; and O_3_—17.79 and 16.23 ppb.

### 3.5. Impact of Mid-Term Air Pollutant Exposure on High-WBC Group

In both the unadjusted and adjusted analyses, mid-term exposure to PM_10_ (30-day: OR = 0.968, 95% CI: 0.937–1.000, *p* = 0.048; 90-day: OR = 0.966, 95% CI: 0.937–0.995, *p* = 0.023) and PM_2.5_ (30-day: OR = 0.970, 95% CI: 0.943–0.998, *p* = 0.035; 90-day: OR = 0.965, 95% CI: 0.938–0.992, *p* = 0.010) were inversely associated with the risk of belonging to the high-WBC count group (Table 5). In the unadjusted analysis, 30- and 90-day exposure to SO_2_ was associated with a lower risk of high WBC count, while 90-day exposure to O_3_ was linked to a higher risk. However, these associations disappeared in the adjusted analysis.

The ORs were calculated per interquartile range increase in the 30-day and 90-day averages for each air pollutant: PM_10_—18.61 and 15.87 μg/m^3^; PM_2.5_—8.61 and 7.29 μg/m^3^; SO_2_—2.44 and 2.33 ppb; NO_2_—17.63 and 17.35 ppb; CO—187.99 and 180.58 ppb; and O_3_—15.47 and 13.64 ppb.

### 3.6. Impact of Long-Term Air Pollutant Exposure on High-WBC Group

In the unadjusted analysis, long-term exposure to PM_2.5_, CO, and O_3_ was associated with a higher risk of belonging to the high-WBC count group (Table 6). In the adjusted analysis, PM_10_ exposure consistently showed a positive association with the high-WBC group across all timeframes (1-year: OR = 1.059, 95% CI: 1.032–1.087, *p* < 0.001; 3-year: OR = 1.067, 95% CI: 1.039–1.096, *p* < 0.001; 5-year: OR = 1.077, 95% CI: 1.044–1.110, *p* < 0.001). Similarly, PM_2.5_ exposure was significantly associated with a higher risk of being in the high-WBC group for 3-year (OR = 1.032, 95% CI: 1.011–1.054, *p* = 0.003) and 5-year periods (OR = 1.036, 95% CI: 1.011–1.062, *p* = 0.005).

NO_2_ (1-year: OR = 1.050, 95% CI: 1.015–1.086, *p* = 0.005; 3-year: OR = 1.046, 95% CI: 1.011–1.082, *p* = 0.009; 5-year: OR = 1.051, 95% CI: 1.010–1.093, *p* = 0.015) and CO (1-year: OR = 1.068, 95% CI: 1.036–1.100, *p* < 0.001; 3-year: OR = 1.072, 95% CI: 1.039–1.106, *p* < 0.001; 5-year: OR = 1.084, 95% CI: 1.044–1.125, *p* < 0.001) demonstrated consistent positive associations with higher WBC counts across all long-term exposure periods. In contrast, O_3_ showed significant associations in the unadjusted analysis, but these associations disappeared after adjustment.

The ORs were calculated per interquartile range increase in the 1-, 3-, and 5-year averages for each air pollutant: PM_10_—8.24, 8.3, and 7.96 μg/m^3^; PM_2.5_—4.57, 3.64, and 3.53 μg/m^3^; SO_2_—1.57, 1.56, and 1.44 ppb; NO_2_—16.45, 16.21, and 16.58 ppb; CO—132.63, 141.12, and 139.82 ppb; and O_3_—5.58, 5.27, and 5.28 ppb.

## 4. Discussion

Our nationwide cross-sectional study in South Korea demonstrated an association between short- and long-term exposure to air pollution and WBC count in the general population. We found that short-term exposure to PM_2.5_, SO_2_, and O_3_ correlated with a decrease in WBC count, while CO and NO_2_ showed positive associations. Mid-term exposure to NO_2_ and CO was positively associated with WBC counts, whereas exposure to PM10, PM_2.5_, and O_3_ was inversely associated. Additionally, long-term exposure to PM_10_, PM_2.5_, NO_2_, and CO was associated with increased WBC counts, while O_3_ showed an inverse association. These findings highlight the pollutant-specific and exposure-duration-dependent effects on WBC counts.

We found that short-term exposure to PM_2.5_ and SO_2_ was negatively associated with WBC counts, while CO and NO_2_ showed positive associations. Previous research on the relationship between short-term air pollutant exposure and WBC count has yielded inconsistent results [16,21,22,23]. A German study found that lag-0 exposure to PM_2.5_ was associated with a decrease in WBC count of −1.6% per IQR change (95% CI: −3.2–0.0) in 57 non-smoking men with coronary heart disease [23]. Rich et al. reported a decrease in WBC count with SO_2_ exposure over lag periods of four to six days in 125 healthy young Chinese adults [22], consistent with our findings. However, Rich et al. also found decreases in WBC counts associated with short-term exposure to CO and NO_2_ over the same lag periods [22], which contrasts with our results showing positive associations for these pollutants. In contrast, a cross-sectional study by Poursafa et al. involving 134 Iranian children and adolescents found a positive correlation between one-week exposure to NO_2_ (β = 0.41) and CO (β = 0.45) and WBC counts, even after adjusting for confounding factors [16], aligning with our findings for these pollutants. Another Chinese study involving 11,035 men aged 22–45 years in Beijing found that short-term exposure to air pollutants over a zero- to three-day lag period had no significant impact on the total peripheral blood WBC count [18]. The observed discrepancies may stem from differences in study populations, geographic regions, pollutant concentrations, and methodological approaches. Our study utilized both linear regression and median-based analyses while accounting for environmental factors associated with air pollution, such as temperature and humidity, to ensure more robust adjustments. These methodological differences highlight the complexity of the relationship and underscore the need for further research to clarify the mechanisms and factors driving the variability in findings.

Although our study did not measure WBC differentials and relied on retrospectively assigned air pollution exposure in a cross-sectional design, it provides insights into the short-term effects of air pollution on WBC counts. Short-term exposure to traffic-related pollutants or PM has been shown to trigger acute airway inflammation, leading to temporary increases in WBC counts, especially neutrophils [19,31]. However, in our study, we observed a significant negative association between same-day exposure to PM_2.5_ and SO_2_ and WBC counts. This finding suggests that these pollutants may induce localized or oxidative stress-related responses that suppress systemic WBC mobilization rather than promote a systemic inflammatory reaction [22,23]. These results align with previous studies reporting decreases in certain WBC subtypes following acute pollutant exposure [19,32,33], highlighting the pollutant-specific and complex nature of short-term air pollution effects on systemic inflammation.

Our study showed that long-term exposure to PM was associated with an increase in WBC counts, similar to a previous study [21]. A study from the United States (NHANES III) reported a significant association between an IQR increase in six-week average PM_10_ exposure and a high WBC count (90th percentile), with an OR of 1.64 (95% CI: 1.17–2.30) [21]. These findings may be attributed to the pro-inflammatory effects of particulate matter. Long-term exposure to PM can trigger oxidative stress and inflammatory responses in the lungs, leading to the release of cytokines such as interleukin-6 (IL-6) and tumor necrosis factor-alpha (TNF-α) [34]. These cytokines stimulate bone marrow activity, resulting in increased white blood cell production and release into circulation [35].

In our study, exposure to NO_2_ increased the risk of being in the increased-WBC group. However, previous studies reported conflicting results [17,18,36]. Yang et al. found no correlation between NO_2_ and WBC counts in a cross-sectional study involving 5816 participants from rural villages in the northern Henan Province, China [17]. Similarly, a study on the adult male population in Beijing, China, also indicated that short-term exposure to NO_2_ was not associated with WBC count [36]. In contrast, Hung et al.’s population-based observational study, which included 10,140 adults, used a multivariate model and reported that an increase in NO_2_ concentration from Q1 to Q3 resulted in a decrease of 432.6 in WBC count [18]. The discrepancies between our findings and those of previous studies may be attributed to differences in exposure assessment methods. While earlier studies relied on NO_2_ levels measured at monitoring stations, our study estimated exposure based on residential addresses, potentially providing a more accurate and individualized assessment.

Our study revealed that prolonged exposure to air pollution, particularly for over one year, was associated with an increase in WBC counts, except in the case of SO_2_ and O_3_. Such long-term exposure to air pollutants can lead to persistent low-grade inflammation in the body [37], stimulating the bone marrow to produce more WBCs over time. Additionally, it is linked to oxidative stress, which further influences immune cell populations [37,38,39]. Oxidative stress generates reactive oxygen species, stimulating the release of pro-inflammatory cytokines, such as IL-6, TNF-α, and IL-1β, and contributing to shifts in immune cell populations [38].

A negative association between SO_2_ exposure and WBC counts was observed in our study. This finding aligns with previous research indicating that SO_2_ exposure can lead to a decrease in WBC counts [17,22,36], particularly neutrophils and monocytes [17]. Additionally, SO_2_ exposure has been linked to genotoxic effects and lymphocyte aberrations [40], further contributing to reduced WBC counts. These findings underscore the need for further research to elucidate the specific pathways through which SO_2_ exposure affects WBC levels and to assess the potential health implications of these changes.

In our study, O_3_ exposure was inversely associated with WBC counts across all time periods, contrasting with the positive associations observed for other pollutants such as PM, CO, and NO_2_. This inverse relationship may be attributed to the unique oxidative properties of O_3_, which can induce bone marrow suppression [41], potentially leading to leukocyte sequestration or apoptosis, thereby reducing circulating WBC counts. Additionally, O_3_ often exhibits a negative correlation with other pollutants, suggesting that areas with higher O_3_ levels may have lower concentrations of PM, CO, and NO_2_, which are typically associated with increased WBC counts. These findings underscore the need for multipollutant models to clarify air pollutants’ combined and individual effects on hematological parameters.

Our study had several limitations. First, the cross-sectional design restricted our ability to establish causality or infer temporal relationships between air pollution exposure and changes in WBC count. To address this limitation, we conducted rigorous statistical analysis and adjusted for confounding variables. However, we acknowledge the possibility of residual confounding due to unmeasured factors. Second, this study was conducted in South Korea, where high levels of air pollutants are common. This may limit the generalizability of our findings to regions with lower pollution levels and different demographic and environmental conditions. Third, we utilized KNHANES data and were unable to obtain additional information such as WBC differentials. Furthermore, we were unable to include other inflammatory markers, such as C-reactive protein, IL-1, or tumor necrosis factor-α, which may have provided a more comprehensive understanding of systemic inflammation, due to the lack of available data in the dataset. Fourth, the analysis assumed that participants stayed in the same location over time, which may introduce bias if residential changes affected actual exposure levels. Lastly, our study did not employ advanced mixture analysis methods, such as Bayesian Kernel Machine Regression or Quantile G-Computation, which are specifically designed to estimate the combined effects of multiple air pollutants. Future studies should consider utilizing these techniques to better characterize the complex interactions among pollutants.

Despite these limitations, our study provides valuable insights into the intricate relationship between air pollution and WBC counts in the general population.

In conclusion, our study in South Korea examining short-, mid-, and long-term impact found that air pollution has complex effects on WBC counts. Our findings emphasize that different pollutants and exposure durations can have varying effects on systemic inflammation, as reflected in WBC levels. Further research is required to understand this relationship and its implications for public health.

## Figures and Tables

**Table 1:** Baseline demographics of total population.

Variable	n = 54,756
Age, years	49.78 ± 0.07
Male	23,663 (43.2%)
Income	
Low	13,203 (24.1%)
Lower-middle	13,725 (25.1%)
Upper-middle	13,889 (25.4%)
High	13,939 (25.5%)
Education	
Elementary or less	12,793 (23.4%)
Middle school	5831 (10.7%)
High school	18,742 (34.2%)
College or higher	17,390 (31.8%)
Ever-smokers	20,982 (38.3%)
Walking days in a week	
0 days	8753 (16.0%)
1 day	3651 (6.7%)
2 days	5444 (9.9%)
3 days	6970 (12.7%)
4 days	4045 (7.4%)
5 days	5706 (10.4%)
6 days	2958 (5.4%)
7 days (everyday)	17,228 (31.5%)
BMI, kg/m^2^	23.76 ± 0.01
WBC, 10^3^/µL	5.91 (2.1)
Hemoglobin level, g/dL	13.9 (2.2)
Platelet count, 10^3^/µL	251 (74)

Values are expressed as mean ± standard error, median (IQR), or number (%), as appropriate. WBC—white blood cell. BMI—body mass index.

**Table 2:** Air pollutant levels across various time periods for the total population.

	PM_10_, μg/m^3^	PM_2.5_, μg/m^3^	SO_2_, ppb	NO_2_, ppb	CO, ppb	O_3_, ppb
On-the-day	50.06 ± 0.10	24.79 ± 0.05	4.83 ± 0.01	25.34 ± 0.06	487.49 ± 0.92	26.66 ± 0.06
7-day	49.75 ± 0.07	24.54 ± 0.04	4.76 ± 0.01	24.45 ± 0.05	482.09 ± 0.73	26.95 ± 0.05
30-day	49.60 ± 0.05	24.43 ± 0.03	4.75 ± 0.01	24.41 ± 0.05	481.14 ± 0.63	26.92 ± 0.05
90-day	49.56 ± 0.05	24.41 ± 0.02	4.77 ± 0.01	24.40 ± 0.05	482.20 ± 0.59	26.90 ± 0.04
1-year	49.91 ± 0.03	24.53 ± 0.02	4.87 ± 0.01	24.55 ± 0.04	487.28 ± 0.39	25.95 ± 0.02
3-year	50.96 ± 0.03	25.14 ± 0.01	5.02 ± 0.01	24.51 ± 0.04	498.02 ± 0.41	24.94 ± 0.02
5-year	50.73 ± 0.03	25.11 ± 0.02	5.00 ± 0.01	24.85 ± 0.05	495.58 ± 0.44	24.89 ± 0.02

Data are expressed as mean ± standard error for continuous variables. PM_10_—particulate matter 10 μm or less in diameter. PM_2.5_—particulate matter 2.5 μm or less in diameter. SO_2_—sulfur dioxide. NO_2_—nitrogen dioxide. CO—carbon monoxide. O_3_—ozone. ppb—parts per billion.

**Table 3:** Correlation between WBC count and air pollutant levels across different time periods.

Variable	Unadjusted		Adjusted *	
	% Change (95% CI)	*p*-Value	% Change (95% CI)	*p*-Value
PM_10_, μg/m^3^				
On-the-day	0.00 (−0.01; 0.01)	0.378	0.00 (−0.01; 0.01)	0.719
7-day	−0.01 (−0.02; 0.01)	0.268	0.00 (−0.01; 0.01)	0.909
30-day	−0.02 (−0.04; 0.00)	0.018	−0.01 (−0.03; 0.01)	0.212
90-day	−0.05 (−0.07; −0.03)	<0.001	−0.03 (−0.05; −0.01)	0.006
1-year	0.01 (−0.03; 0.04)	0.682	0.09 (0.06; 0.13)	<0.001
3-year	0.00 (−0.04; 0.03)	0.871	0.12 (0.08; 0.15)	<0.001
5-year	−0.02 (−0.06; 0.02)	0.268	0.13 (0.08; 0.17)	<0.001
PM_2.5_, μg/m^3^				
On-the-day	−0.02 (−0.04; 0.00)	0.041	−0.02 (−0.04; 0.0)	0.022
7-day	−0.03 (−0.06; −0.01)	0.011	−0.03 (−0.06; 0.0)	0.020
30-day	−0.07 (−0.10; −0.03)	<0.001	−0.07 (−0.11; −0.03)	<0.001
90-day	−0.12 (−0.17; −0.08)	<0.001	−0.12 (−0.16; −0.08)	<0.001
1-year	−0.05 (−0.11; 0.01)	0.129	−0.02 (−0.08; 0.04)	0.504
3-year	0.07 (0.00; 0.13)	0.043	0.15 (0.08; 0.21)	<0.001
5-year	0.11 (0.03; 0.19)	0.007	0.22 (0.15; 0.30)	<0.001
SO_2_, ppb				
On-the-day	−72.36 (−87.11; −40.71)	0.001	−58.01 (−80.23; −10.82)	0.024
7-day	−87.23 (−94.81; −68.59)	<0.001	−72.31 (−88.53; −33.14)	0.004
30-day	−88.41 (−95.59; −69.58)	<0.001	−72.46 (−89.34; −28.81)	0.008
90-day	−93.09 (−97.51; −80.83)	<0.001	−76.35 (−91.16; −36.72)	0.004
1-year	−79.25 (−93.68; −31.9)	0.010	26.14 (−59.66; 294.43)	0.690
3-year	−54.78 (−86.42; 50.59)	0.196	156.31 (−18.92; 710.26)	0.109
5-year	−38.47 (−84.48; 143.88)	0.489	253.04 (−5.72; 1222.0)	0.061
NO_2_, ppb				
On-the-day	9.78 (−6.23; 28.53)	0.246	38.46 (15.29; 66.30)	<0.001
7-day	6.91 (−11.54; 29.20)	0.489	43.85 (17.38; 76.28)	<0.001
30-day	10.70 (−9.55; 35.48)	0.324	49.89 (20.63; 86.25)	<0.001
90-day	1.030 (−17.91; 24.34)	0.923	34.45 (8.36; 66.81)	0.007
1-year	18.22 (−5.89; 48.51)	0.150	71.24 (35.9; 115.76)	<0.001
3-year	10.00 (−12.65; 38.52)	0.418	55.73 (23.3; 96.70)	<0.001
5-year	−17.08 (−36.39; 8.10)	0.166	48.2 (13.21; 94.01)	0.004
CO, ppb				
On-the-day	0.58 (−0.47; 1.65)	0.278	1.93 (0.74; 3.14)	0.001
7-day	0.02 (−1.30; 1.35)	0.562	1.85 (0.32; 3.40)	0.017
30-day	0.45 (−1.07; 2.00)	0.126	3.28 (1.35; 5.24)	0.001
90-day	−1.28 (−2.89; 0.36)	0.071	0.96 (−0.88; 2.83)	0.310
1-year	2.32 (−0.19; 4.89)	0.265	9.56 (6.89; 12.30)	<0.001
3-year	1.36 (−1.02; 3.80)	0.244	10.08 (7.43; 12.80)	<0.001
5-year	−1.70 (−4.49; 1.17)	0.278	8.56 (5.39; 11.83)	<0.001
O_3_, ppb				
On-the-day	6.72 (−9.79; 26.25)	0.448	−35.4 (−47.70; −20.21)	<0.001
7-day	5.66 (−12.18; 27.12)	0.560	−45.11 (−56.45; −30.81)	<0.001
30-day	18.81 (−2.66; 45.01)	0.090	−40.69 (−54.61; −22.49)	<0.001
90-day	47.45 (18.79; 83.02)	<0.001	−18.98 (−41.15; 11.56)	0.197
1-year	419.27 (246.44; 678.31)	<0.001	−15.8 (−47.28; 34.47)	0.472
3-year	547.58 (301.71; 943.92)	<0.001	−32.34 (−61.27; 18.22)	0.170
5-year	614.44 (304.49; 1161.91)	<0.001	−60.7 (−79.78; −23.60)	0.006

The WBC count was logarithmically transformed, and the results were expressed as percentage changes in WBC count per unit increase in each pollutant. PM_10_—particulate matter 10 μm or less in diameter. PM_2.5_—particulate matter 2.5 μm or less in diameter. SO_2_—sulfur dioxide. NO_2_—nitrogen dioxide. CO—carbon monoxide. O_3_—ozone. ppb—parts per billion. * Adjusted for examination year, sex, age, income, education level, hemoglobin level, platelet count, smoking status, walking days in a week, temperature, wind speed, humidity, precipitation, wind direction, solar radiation, and surface pressure.

**Table 4:** Impact of short-term air pollutant exposure on high-WBC group.

Variable	Unadjusted		Adjusted *	
	OR (95% CI)	*p*-Value	OR (95% CI)	*p*-Value
PM_10_, μg/m^3^				
On-the-day	0.988 (0.968; 1.008)	0.229	0.976 (0.952; 0.999)	0.045
7-day	0.991 (0.970; 1.012)	0.387	0.991 (0.965; 1.017)	0.48
PM_2.5_, μg/m^3^				
On-the-day	0.975 (0.955; 0.995)	0.014	0.965 (0.941; 0.988)	0.004
7-day	0.980 (0.959; 1.002)	0.073	0.986 (0.961; 1.011)	0.272
SO_2_, ppb				
On-the-day	0.979 (0.964; 0.995)	0.010	0.979 (0.961; 0.998)	0.027
7-day	0.972 (0.956; 0.989)	0.001	0.982 (0.962; 1.001)	0.067
NO_2_, ppb				
On-the-day	1.012 (0.989; 1.035)	0.315	1.009 (0.977; 1.042)	0.577
7-day	1.008 (0.983; 1.033)	0.528	1.014 (0.982; 1.047)	0.400
CO, ppb				
On-the-day	1.005 (0.987; 1.023)	0.598	1.010 (0.985; 1.035)	0.437
7-day	0.999 (0.981; 1.018)	0.948	1.008 (0.982; 1.035)	0.564
O_3_, ppb				
On-the-day	1.011 (0.988; 1.033)	0.354	0.977 (0.944; 1.011)	0.179
7-day	1.009 (0.986; 1.031)	0.446	0.973 (0.94; 1.007)	0.122

PM_10_—particulate matter 10 μm or less in diameter. PM_2.5_—particulate matter 2.5 μm or less in diameter. SO_2_—sulfur dioxide. NO_2_—nitrogen dioxide. CO—carbon monoxide. O_3_—ozone. OR—odds ratio. CI—confidence interval. ppb—parts per billion. * Adjusted for examination year, sex, age, income, education level, hemoglobin level, platelet count, smoking status, walking days in a week, temperature, wind speed, humidity, precipitation, wind direction, solar radiation, and surface pressure.

**Table 5:** Impact of mid-term air pollutant exposure on high-WBC group.

Variable	Unadjusted		Adjusted *	
	OR (95% CI)	*p*-Value	OR (95% CI)	*p*-Value
PM_10_, μg/m^3^				
30-day	0.977 (0.953; 1.001)	0.060	0.968 (0.937; 1.000)	0.048
90-day	0.957 (0.935; 0.981)	<0.001	0.966 (0.937; 0.995)	0.023
PM_2.5_, μg/m^3^				
30-day	0.969 (0.947; 0.991)	0.007	0.970 (0.943; 0.998)	0.035
90-day	0.957 (0.933; 0.976)	<0.001	0.965 (0.938; 0.992)	0.010
SO_2_, ppb				
30-day	0.972 (0.955; 0.989)	0.002	0.983 (0.963; 1.003)	0.102
90-day	0.970 (0.953; 0.987)	0.001	0.987 (0.967; 1.008)	0.218
NO_2_, ppb				
30-day	1.014 (0.988; 1.041)	0.295	1.019 (0.985; 1.055)	0.282
90-day	1.006 (0.980; 1.034)	0.645	1.014 (0.981; 1.049)	0.411
CO, ppb				
30-day	1.008 (0.986; 1.029)	0.486	1.018 (0.986; 1.052)	0.270
90-day	0.995 (0.973; 1.017)	0.626	1.005 (0.975; 1.036)	0.745
O_3_, ppb				
30-day	1.016 (0.993; 1.039)	0.184	0.976 (0.939; 1.014)	0.211
90-day	1.031 (1.008; 1.053)	0.007	1.011 (0.970; 1.053)	0.614

PM_10_—particulate matter 10 μm or less in diameter. PM_2.5_—particulate matter 2.5 μm or less in diameter. SO_2_—sulfur dioxide. NO_2_—nitrogen dioxide. CO—carbon monoxide. O_3_—ozone. OR—odds ratio. CI—confidence interval. ppb—parts per billion. * Adjusted for examination year, sex, age, income, education level, hemoglobin level, platelet count, smoking status, walking days in a week, temperature, wind speed, humidity, precipitation, wind direction, solar radiation, and surface pressure.

**Table 6:** Impact of long-term air pollutant exposure on high-WBC group.

Variable	Unadjusted		Adjusted *	
	OR (95% CI)	*p*-Value	OR (95% CI)	*p*-Value
PM_10_, μg/m^3^				
1-year	1.006 (0.985; 1.027)	0.574	1.059 (1.032; 1.087)	<0.001
3-year	0.998 (0.978; 1.020)	0.882	1.067 (1.039; 1.096)	<0.001
5-year	0.993 (0.969; 1.017)	0.551	1.077 (1.044; 1.11)	<0.001
PM_2.5_, μg/m^3^				
1-year	0.993 (0.973; 1.014)	0.533	1.018 (0.993; 1.043)	0.161
3-year	1.016 (0.998; 1.034)	0.081	1.032 (1.011; 1.054)	0.003
5-year	1.026 (1.005; 1.047)	0.016	1.036 (1.011; 1.062)	0.005
SO_2_, ppb				
1-year	0.990 (0.976; 1.003)	0.141	1.005 (0.989; 1.021)	0.537
3-year	0.997 (0.983; 1.011)	0.637	1.006 (0.99; 1.022)	0.496
5-year	1.004 (0.989; 1.019)	0.608	1.016 (0.999; 1.033)	0.068
NO_2_, ppb				
1-year	1.024 (0.996; 1.053)	0.096	1.05 (1.015; 1.086)	0.005
3-year	1.016 (0.989; 1.045)	0.249	1.046 (1.011; 1.082)	0.009
5-year	0.994 (0.962; 1.027)	0.710	1.051 (1.01; 1.093)	0.015
CO, ppb				
1-year	1.025 (1.001; 1.051)	0.045	1.068 (1.036; 1.1)	<0.001
3-year	1.019 (0.994; 1.045)	0.135	1.072 (1.039; 1.106)	<0.001
5-year	1.002 (0.972; 1.032)	0.902	1.084 (1.044; 1.125)	<0.001
O_3_, ppb				
1-year	1.057 (1.039; 1.074)	<0.001	0.989 (0.965; 1.014)	0.382
3-year	1.061 (1.041; 1.081)	<0.001	0.984 (0.958; 1.011)	0.239
5-year	1.061 (1.037; 1.085)	<0.001	0.981 (0.951; 1.013)	0.245

PM_10_—particulate matter 10 μm or less in diameter. PM_2.5_—particulate matter 2.5 μm or less in diameter. SO_2_—sulfur dioxide. NO_2_—nitrogen dioxide. CO—carbon monoxide. O_3_—ozone. OR—odds ratio. CI—confidence interval. ppb—parts per billion. * Adjusted for examination year, sex, age, income, education level, hemoglobin level, platelet count, smoking status, walking days in a week, temperature, wind speed, humidity, precipitation, wind direction, solar radiation, and surface pressure.

## Data Availability

All data used in this study are publicly accessible through the KNHANES website (http://knhanes.cdc.go.kr).

## Supplementary Materials

The following supporting information can be downloaded at: https://www.mdpi.com/article/10.3390/jcm13237402/s1, Supplementary Table S1. Survey design and weighting details; Supplementary Table S2. Median (IQR) levels of air pollutants across various time periods for the total population; Supplementary Table S3. Environmental factors at day 0; Supplementary Table S4. Correlation between air pollutants at day 0.

## Abbreviations

The following abbreviations are used in this manuscript:

WBC	white blood cells
KNHANES	Korean National Health and Nutrition Examination Survey
PM_10_	particulate matter 10 μm or less in diameter
PM_2.5_	particulate matter 2.5 μm or less in diameter
SO_2_	sulfur dioxide
NO_2_	nitrogen dioxide
O_3_	ozone
CO	carbon monoxide
OR	odds ratio
CI	confidence interval
IQR	interquartile range
ppb	parts per billion
