# Supplementary material for: The Association Between Air Pollution Exposure and White Blood Cell Counts: A Nationwide Cross-Sectional Survey in South Korea
Source: J Clin Med. 2024 Dec 5;13(23):7402. doi: 10.3390/jcm13237402 (PMC11642379; doi:10.3390/jcm13237402)
Supplement: Supplementary file 1 [file jcm-13-07402-s001.zip › jcm-3334769-supplementary.pdf]

The Association between Air Pollution Exposure and White Blood Cell Counts: A South Korea Nationwide Cross-sectional Survey

Supplementary Table S1. Survey design and weighting details

Study design
This study used data from the 2007–2019 Korea National Health and Nutrition Examination Survey (KNHANES), which applies a stratified, multistage probability sampling design to ensure national representativeness. The sampling weights provided by KNHANES were calculated to adjust for several factors: (1) unequal selection probabilities due to the complex sampling design, (2) participant non-response to account for missing data within sampled clusters or strata, and (3) post-stratification adjustments to align the sample distribution with the total Korean population based on demographic factors such as age, sex, and region.<br><br>Specifically, the weights (wt) were provided for three survey components—health interview, health behavior, and health examination—and were combined when conducting analyses that used variables from multiple components. These weights ensure that the estimates from the sample accurately represent the population while maintaining consistency across the different survey components. The combined weights were applied directly in the survey design to reflect both the probability of selection and post-stratification adjustments, making them essential for unbiased population-level inference.<br><br>The survey package in R (v4.1-1, Lumley, 2020) was used to implement these adjustments. Below is an example of the code specifying the design:

```
library(survey)

# Define survey design
design <- svydesign(
   id = ~PSU,                  # Primary sampling units (clusters)
   strata = ~STRATA,      # Stratification variables
   weights = ~wt,           # Combined sampling weights from KNHANES
   data = final_dataset, # Final dataset containing 2007–2019 data
   nest = TRUE                # Indicating a nested survey design
)

# Example of weighted analysis
# Calculate weighted mean
svymean(~variable_of_interest, design)

# Example of weighted logistic regression
svyglm(outcome ~ predictor1 + predictor2, design = design, family = quasibinomial())
```

Supplementary Table S2. Median (IQR) levels of air pollutants across various time periods in total population

	PM_{10}, μg/m^3	$PM_{2.5}$, μg/m^3	SO_2, ppb	NO_2, ppb	CO, ppb	O_3, ppb
On the day	46.22 (26.61)	22.39 (14.84)	4.17 (2.8)	22.67 (19.65)	443.77 (229)	25.16 (17.79)
7-day	47.85 (21.2)	23.43 (11.11)	4.24 (2.53)	23.19 (17.54)	456.14 (192.26)	25.49 (16.23)
30-day	49.33 (18.61)	23.98 (8.61)	4.32 (2.44)	23.56 (17.63)	457.45 (187.99)	25.55 (15.47)
90-day	49.18 (15.87)	24.17 (7.29)	4.36 (2.33)	23.64 (17.35)	460.53 (180.58)	25.76 (13.64)
1-year	49.46 (8.24)	24.23 (4.57)	4.63 (1.57)	22.9 (16.45)	501.9 (132.63)	24.87 (5.58)
3-year	50.5 (8.3)	24.9 (3.64)	4.77 (1.56)	22.86 (16.21)	516.83 (141.12)	24.31 (5.27)
5-year	50.42 (7.96)	24.95 (3.53)	4.74 (1.44)	23.36 (16.58)	515.74 (139.82)	24.43 (5.28)

Data are expressed as median (interquartile range, IQR) for continuous variables. PM_{10}, particulate matter 10 micrometers or less in diameter. $PM_{2.5}$, particulate matter 2.5 micrometer or less in diameter. SO_2, sulfur dioxide. NO_2, nitrogen dioxide. CO, carbon monoxide. O_3, ozone. ppb, parts per billion.

Supplementary Table S3. Environmental factors at day 0

Variable	**Levels**
Temperature, ℃	12.65 ± 0.04
Wind speed, m/s	2.67 ± 0.01
Humidity, %	67.71 ± 0.06
Precipitation, cm/hr	0.01 ± 0.002
Wind direction, °	191.32 ± 0.34
Solar radiation, W/m^2	178.86 ± 0.35
Surface pressure, hPa	1003.64 ± 0.06

Data are expressed as mean ± standard error for continuous variables.

Supplementary Table S4. Correlation between air pollutants at day 0.

		PM_{10},	$PM_{2.5}$,	SO_2	NO_2	CO	O_3
PM_{10},	r	1.000	0.892	0.552	0.524	0.686	-0.101
	p-value		<0.001	<0.001	<0.001	<0.001	<0.001
$PM_{2.5}$,	r		1.000	0.475	0.411	0.619	-0.073
	p-value			<0.001	<0.001	<0.001	<0.001
SO_2	r			1.000	0.499	0.533	-0.242
	p-value				<0.001	<0.001	<0.001
NO_2	r				1.000	0.709	-0.462
	p-value					<0.001	<0.001
CO	r					1.000	-0.407
	p-value						<0.001
O_3	r						1.000
	p-value						

$PM_{2.5}$, particulate matter 2.5 micrometer or less in diameter. SO_2, sulfur dioxide. NO_2, nitrogen dioxide. CO, carbon monoxide. O_3, ozone. ppb, parts per billion
